# Atlanta Academy of Medicine

**Published:** 1874-04

**Authors:** James B. Baird

**Affiliations:** Reporter


### Atlanta Academy of Medicine.

---

JAMES B. BAIRD, M.D., REPORTER.

---

ATLANTA, GA., February 9, 1874.

Dr. W. S. Armstrong, President, presiding.

Dr. Battey stated that the case of ovarian tumor reported by him two weeks ago, had been examined by a gynæcologist of Augusta. The diagnosis was confirmed. The opinion expressed in favor of removal concurred in, and the proposed operation endorsed.

Dr. Connally reported the case of a large, stout, mulatto woman, æt. twenty-eight, with great effusion into subcutaneous cellular tissue; has been affected thus for six weeks, but recently the effusion has largely increased; no cardiac lesions; urine three ounces in twenty-four hours; intensely albuminous, the usual test solidifies the specimen; microscope reveals the presence of epithelial casts; intensely acid; specific gravity 1,051. The amount increased within a few days to six ounces; is taking hydrogogue cathartics.

Dr. Battey remarked that this case reminded him of the case of an old gentleman, æt. sixty, who had had vavlular disease of the heart for years; urine at times albuminous; no microscopical examination was made; symptoms indicated subacute Bright's disease. Under the treatment of diuretics, digitalis diaphoretics, stimulants, bromide of potassium, etc., etc., he grew worse; on one occasion he remained with him during the night expecting him to die. He was comatose, poisoned, he supposed, on account of the failure of elimination upon the part of the kidneys; urine and feces passed involuntarily; treatment suspended from necessity, as there was inability to swallow. In a few hours he began to improve. He was let alone, and he recovered, and he is now in a good state of general health. Improvement was not preceded by any very free action of the bowels or kidneys.

Dr. Taliaferro had used, in albuminuria and uræmia of pregnancy, bromide of potassium with great satisfaction. Dr. Thomas, of Savannah, reported, at a recent meeting of the State Medical Association, the successful treatment of uterine convulsions by the same remedy. He also used it in threatened convulsions. Several years ago was called to see a pregnant woman in the absence of her attending physician; found her suffering to such an extent from albuminuria, that for her safety, he advised premature labor. He ordered, however, bromide of potassium twenty to thirty grains every four hours. The albumen in her urine rapidly decreased and finally disappeared, and she was delivered, at full time, of a healthy child.

Dr. DuPre, a number of years ago, met with a gentleman, æt. sixty, suffering from anasarca, the result of very serious organic disease of the heart, the right side being hypertrophied and dilated. The patient had passed through the usual treatment for anasarca without relief, and he determined to try the full eliminative value of calomel. Vegetable tonics were combined with the mercury, which was continued until ptyalism was produced, when the daily amount was diminished. The anasarca, as well as a considerable effusion in the pleura and peritoneum rapidly diminished, and almost entire relief was obtained. The mercury was discontinued, and in a few weeks all of the unpleasant symptoms returned. The same treatment was again instituted with similar results. The patient improved sufficiently to attend to business. He passed from under his observation soon after, and has since died. Has seen the urine *diminish* under the use of diuretics; believes the kidneys to be in a state of hyperæmia and that this class of remedies is not indicated; should expect advantage from cups and a blister over the region of the kidneys.

Dr. DeWitt thought that generally the most successful plan of treatment in this class of cases consisted in the use of digitalis and tincture of iron.

Dr. Battey regards digitalis as a valuable diuretic; considers much of that in market utterly worthless. Thinks digitalis and ergot should not be officinal in the form of fluid extracts; the necessary manipulation to which they are subjected often destroys their value. Prefers the infusion of the leaves of digitalis and those not pressed into cakes, but loose and properly dried. The same remark applies to ergot, he decidedly prefers the ergot powdered extemporaneously and given in infusion. Three or four years ago was asked to see a gentleman in consultation, who had had a diseased heart for a year, with anasarca. The attending physician had exhausted all the means at his command without any benefit to the patient; among them the ordinary preparations of digitalis and hydrogogue cathartics. He ordered an infusion of digitalis, prepared as above described. In three weeks the dropsy was gone and a very marked general improvement was observed. An occasional dose of the digitalis kept down the anasarca. The patient was very sanguine of a complete recovery, notwithstanding he was warned that he had a serious and necessarily fatal disease of the heart.

Dr. John G. Westmoreland reported a similar case, which had been treated with the common preparations of digitalis without benefit. He gave the substance in the form of pill, with mercury, and great improvement followed. Digitalis is a tonic to the kidneys, and acts without producing either hyperæmic or anæmic condition of the organs. It imparts vigor to the kidneys without exciting them. Digitalis also acts on the heart as a tonic, and not as a sedative. He once thought the so-called cumulative effect dangerous; was afraid to give ten drops of the tincture. It has been demonstrated, however, that a half ounce may be given, and repeated, without poisonous effects. He usually gives one or two grains of the powder, continued as long as the necessity for it exists. Does not believe in its cumulative effects.

Dr. Baird inquired in what way bromide of potassium exerted a beneficial influence in such cases, and why did Dr. W. combine mercury with the digitalis in his case.

Dr. Westmoreland replied that he gave the mercury to increase the secretion of bile.

Dr. Taliaferro was inclined to the belief that the beneficial effects of bromide of potassium were due to its sedative action.

Dr. Battey remarked, in reference to the poisonous action of digitalis, he had seen unpleasant effects only in one case, and they were momentary, occurring in a patient under the following circumstances: He had prescribed the infusion for an old gentleman suffering from obstructive valvular lesions of the heart, and consequently congestion of the lungs upon exercise. He, on one occasion, by accident, took a double dose, which very much depressed the pulse and produced other alarming symptoms. This condition continued for an hour, when the untoward symptoms passed off, and very decided relief followed, patient feeling much better than after an ordinary dose. The good effect of bromide of potassium is probably explained in two ways, *i.e.*, by diminishing the caliber of the blood vessels, and thus lessening the supply of blood to the brain, and by obtunding the sensibility of the brain, warding off convulsions and tiding the patient over the critical period. He also believes that, by balancing, as it were, the nervous system, the renal secretion is promoted.

Dr. Armstrong stated that the case of meningitis reported by him two weeks ago terminated fatally the next day. Has since that time had another fatal case of the same disease in a child æt. six or eight. A peculiarity of the last case was tumefaction, extending along the course of one of the sterno-cleido mastoid muscles. Observed this same feature in a fatal case of meningitis last winter. Has met with it during the present winter in cases of ordinary influenza. Saw a case a few days ago of depression of one of the cranial bones. A boy, æt. six or eight, was lying on the ground, when a bale of cotton fell on his head, crushing in the upper border of the temporal bone to an extent equal to the thickness of the skull. When first seen the symptoms of concussion were present; he was sleeping, and pulse very weak. The circulation soon became re-established, and the next day the pulse was 112. Ordered veratrum viride sufficient to maintain pulse at 90. As he has continued to improve without an unfavorable symptom, he determined not to interfere with the depressed bone.

Dr. Battey expressed himself as decidedly of the opinion that in such cases circumstances that would justify surgical interference must be imperative. There is a single exception; in punctured fractures the trephine should be used.

Dr. DuPre indorsed the views expressed by Dr. Battey.

Dr. Battey reported progress on a case originally reported by Dr. W. F. Westmoreland, October 27, of a young lady suffering from an extremely painful tumor of the right mammary gland, for which electricity was used with signal and immediate relief. He recently learned from the patient that the paroxysms of pain are not now confined to the breast, and are not so severe. She says they are "general—all over." She has abandoned the use of the battery, as its remedial effects seem to have been lost; and as the pain is "all over," she does not know where to apply it. Her menstrual function is not perfect; regular but scanty. After the positive current, which was first resorted to, began to lose its effect, the negative was substituted, with equally satisfactory results at first, but it soon wore out, and neither then afforded relief. He is of the opinion that the sole benefit was due simply to the nervous shock, and when the system became gradually accustomed to this it ceased to produce any perceptible effect.

---

Atlanta, Ga., February 16, 1874.

Dr. W. S. Armstrong, President, presiding.

Dr. Battey said, apropos to the discussion at the last meeting, that he was called to see a patient the following day, eight months pregnant, suffering from anasarca and difficult respiration; slight cerebral disturbance, urine scanty—about two or three ounces in twenty-four hours; bowels not constipated; was apprehensive of convulsions. Next day urine somewhat more abundant; no albumen. Ordered bromide of potassium; twenty grains every two hours, for three or four doses, to quiet the nervous system, and dessert-spoonful of the infusion digitalis three times a day. In twenty-four hours the improvement was marked; in sixty hours the anasarca was almost entirely gone; the kidneys responding promptly and freely.

Dr. John G. Westmoreland reported the following case: Girl aged eight or ten; pain in back; experienced great difficulty in moving about; soon became entirely helpless; is unable to move or sit up, from pain in the spinal column. This condition has continued for three weeks. Cups and a blister afforded no relief. There is no fever; excessive tenderness over the whole spine.

Dr. Battey had a parallel case about two years ago; immediate relief was had upon the expulsion of worms from the intestinal canal.

Dr. Stout referred to a similar case in a boy æt. ten; suffered great pain in spine; limbs drawn up. Was treated without benefit for several months for rheumatism; suspected worms. The action of calomel and turpentine confirmed the diagnosis, with entire relief to the patient.

Dr. Battey stated that a correspondent desired the opinion of the Academy in regard to the treatment of nasal catarrh; also as to the nasal douche.

Dr. Logan has no confidence in any proposed mode of treatment; has had considerable experience in those cases, but no permanent success.

Dr. Armstrong called attention to the difference between simple catarrh and ozœna dependent upon carious bone. No expectation of benefit from treatment in the latter disease. He adopted the use of the nasal douche some years ago; has seen great pain result from the employment of medicinal agents of the strength advised by some. In one case inflammation of the internal ear followed the treatment, and it may have been caused by it. Has almost entirely abandoned the use of carbolic acid, nitrate of silver, sulphate of copper, etc. Now uses simply a solution of common salt in water. Has seen several cases cured; only, however, when the disease was confined to the schneiderian membrane; the bones were not affected. Applies the salt and water by means of the nasal douche, and has never seen any ill effect. The application is made one, two or three times daily, and it should be continued for a considerable time after the disappearance of the symptoms.

Dr. Stout had been informed by a number of surgeons in different parts of the country, that they regarded the nasal douche as a dangerous instrument, and that serious consequences sometimes followed the retention of the fluid in the internal ear.

Dr. Armstrong replied that no doubt such cases do occur, but thinks they are due to the too great strength and the irritating character of the solutions employed.

Dr. Battey's observations corresponded with Dr. Armstrong's.

Has employed the douche largely for years, using a solution containing one teaspoonful of common salt to a pint of warm water. He is in the habit of giving at the same time small doses of bichloride of mercury and iodide of potassium. His cases always improve, but has never seen a single one make a *permanent* recovery. He occasionally adds one grain of carbolic acid, sometimes increased to five grains to the pint solution of salt. Years ago he used a solution of permanganate of potassa of half the strength commonly directed, and found it unbearable.

Dr. Calhoun remarked that while in Europe he assisted at two clinics, one in Berlin, and one in Vienna. He used the nasal douche daily, employing warm water containing a small quantity of common salt. Has never seen a single bad result, and the surgeons there express themselves surprised at the experience of American surgeons, many of whom have reported unfavorably, and abandoned the use of the instrument altogether. In the treatment of ozœna, after cleansing out the nose a saturated solution of nitrate of silver is injected by means of a syringe into one nostril and allowed to escape from the other. Has seen two hundred and eighty grains to the ounce injected in this way; immediately neutralized, however, by common salt and water. Does not remember to have seen a perfect cure; there was no constitutional treatment; absolutely local. There is no objection to fluid gaining access to the internal ear. The ustachian tube is daily injected in the treatment of diseases of the middle ear. The injection escapes or is absorbed and no harm is done.

Adjourned.

---

Atlanta, Ga., February 23, 1874.

Dr. W. S. Armstrong, President, presiding.

Dr. J. G. Westmoreland reported progress on the case reported by him at the last meeting, of spinal irritation in a girl. At Dr. Battey's suggestion, he administered an anthelmintic, santonine, followed by oil and turpentine without effect; then ordered calomel gr. x, santonin, oil and turpentine; this was followed by a discharge of intestinal worms, but no improvement in the general symptoms.

Dr. Battey stated that the case of anasarca, during pregnancy, reported by him, continued to do well, the anasarca not having returned. On Friday night the patient was delivered of an eight months' child after an easy, natural labor.

An extract was read from a letter from a gentleman, residing in New York, stating that Dr. Sims had said that Dr. Battey had been followed in his operation of "Normal Ovariotomy" in two cases: one in Mobile, and one in New York. The latter was operated on in November, and on the day of writing was discharged perfectly well and had returned home to get married.

Dr. J. G. Westmoreland had under observation a female suffering from some uterine disorder. About a month ago a tumor, evidently a collection of fluid, was observed on the buttock, and extending from it to the ovary of the same side, was a tender streak. The tumor was punctured and about two ounces of a slightly colored watery fluid was discharged. Recovery was complete.

Dr. Battey reported the case of a little girl, æt. five, who applied to him with a "lump on her head." He made a casual examination, and concluded that it was an ordinary cystic tumor of the scalp, and advised that it be let alone for the present. About three weeks ago an elder sister of the child said there was a black spot on the lump, and that it moved; so, compressing the tumor between her thumbs, a worm, three-fourths of an inch in length and about the size of a crow's quill, popped out, followed by a small quantity of ill-conditioned pus. The grub has a black head and a yellow body, with annular markings of black. His opinion is that it is the same condition as that known as wolves in cattle, and that it originated in this case as it does in animals, by the deposit of an egg through a puncture of the skin, by a fly that has a proboscis resembling the point of a hypodermic syringe. The egg being deposited in the fall, by spring it has developed into a grub or "wolf." This affection in animals is not confined to this country, but is met with in Europe. He regards the case reported unique, he never having heard or read of a similar one. The rather smaller size of the worm than usual was accounted for on the ground of the delicacy of the child's skin.

---

A POW-WOW doctress in Nazareth, Pa., recently undertook to bleed a lady for an epileptic fit. Unfortunately she opened an artery instead of the vein, and the patient died before assistance could be obtained.

A POPULAR doctor in Chicago was presented with a silver-mounted skeleton, on New Year's, by his admiring patients.

